# Atraumatic Splenic Rupture Caused by Splenic Vein Thrombosis in a Patient With Chronic Pancreatitis

**DOI:** 10.7759/cureus.107211

**Published:** 2026-04-17

**Authors:** Ryoichi Takenaka

**Affiliations:** 1 Department of Surgery, Uwajima City Hospital, Uwajima, JPN

**Keywords:** atraumatic splenic rupture, case report, chronic pancreatitis, splenectomy, splenic complications, splenic vein thrombosis

## Abstract

Atraumatic splenic rupture is a rare but potentially life-threatening complication of pancreatic disease. This report describes the case of a 62-year-old man with a prior history of chronic pancreatitis related to alcoholic pancreatitis who presented with left upper abdominal pain without any history of trauma. Contrast-enhanced computed tomography demonstrated splenomegaly, a large splenic laceration, and a perisplenic hematoma without active contrast extravasation, along with a pancreatic cystic lesion and narrowing of the splenic vein. Magnetic resonance imaging showed heterogeneous signal intensity within the spleen, and a neoplastic lesion could not be excluded. On admission, serum amylase was mildly elevated at 149 U/L, HbA1c was 4.8%, and formal pancreatic exocrine function testing had not been performed before surgery. Because he was hemodynamically stable, conservative management was initially selected. However, persistent diagnostic uncertainty and concern for re-bleeding led to surgery on hospital day 9. Distal pancreatectomy with splenectomy and partial diaphragmatic resection was performed. Intraoperatively, the pancreas was indurated, consistent with chronic inflammatory change. Histopathological examination of the resected distal pancreas demonstrated chronic pancreatitis with fibrosis, mild chronic inflammatory cell infiltration, fat necrosis, pseudolobular formation, and focal acute inflammation, with no evidence of malignancy. The final diagnosis was atraumatic splenic rupture secondary to splenic vein thrombosis associated with chronic pancreatitis. This case highlights the need to consider splenic complications in patients with chronic pancreatitis who present with left upper quadrant pain and supports surgical management when malignancy cannot be excluded or when the risk of re-bleeding remains a concern.

## Introduction

Atraumatic splenic rupture is an uncommon clinical entity and accounts for less than 1% of all splenic ruptures. In a large systematic review, Renzulli et al. analyzed 845 patients and found that 93% of cases were associated with an underlying pathological process rather than being idiopathic, with an overall mortality rate of 12.2%. Reported causes include hematologic disorders, infections, malignancies, inflammatory conditions, and pancreatic disease. Although splenic complications are recognized in chronic pancreatitis, splenic rupture remains rare and may be overlooked because the presenting symptoms are often nonspecific [1-3].

The close anatomical relationship between the pancreatic tail and the splenic hilum helps explain why pancreatic inflammation can involve the splenic parenchyma and splenic vessels. Proposed mechanisms of splenic injury in pancreatitis include direct enzymatic damage, rupture of a pancreatic pseudocyst into the spleen, splenic infarction and necrosis, and venous outflow obstruction caused by splenic vein thrombosis, leading to congestion and increased intrasplenic pressure [2,4-6]. A case of atraumatic splenic rupture caused by splenic vein thrombosis with occlusion associated with chronic pancreatitis is described here, in which pathological examination was essential for confirming the diagnosis and excluding malignancy.

This article was previously presented as a poster at the 84th Annual Congress of Japan Surgical Association, held from November 24 to 26, 2022.

## Case presentation

A 62-year-old man was transferred to this hospital because of persistent epigastric and left upper quadrant pain that had begun 10 days earlier. He reported no recent history of trauma. At a local clinic, abdominal ultrasonography demonstrated fluid collection around the spleen, and computed tomography revealed splenomegaly, splenic laceration, an acute perisplenic hematoma, and intraperitoneal fluid collection. He was therefore referred for further management.

His medical history was significant for chronic pancreatitis related to alcoholic pancreatitis. He had no known drug allergies and was not taking any regular medications. He was a former heavy alcohol user and currently consumed approximately 500 mL of beer per week, whereas about 10 years earlier, he had reportedly consumed as much as 1,500 mL per day. He did not smoke and was independent in activities of daily living.

On admission, he was alert and oriented. His body temperature was 38°C, his blood pressure was 133/90 mmHg, his pulse rate was 87 beats/min, and his respiratory rate was 22 breaths/min. Abdominal examination revealed tenderness and rebound tenderness in the left upper quadrant, without muscular guarding. Chest radiography and electrocardiography showed no remarkable abnormalities.

Laboratory findings on admission are summarized in Table 1. The patient had anemia, hypoalbuminemia, and elevated C-reactive protein, lactate dehydrogenase, gamma-glutamyl transferase, amylase, and soluble interleukin-2 receptor levels. Serum amylase was 149 U/L. HbA1c was 4.8%, and no clear endocrine dysfunction was identified at presentation. The white blood cell count was within the reference range, and renal function was preserved. Formal pancreatic exocrine function testing had not been performed before surgery.

**Table 1:** Laboratory findings on admission

Test name	Unit	Observed value	Normal range
White blood cell count	×10^3^/μL	6.31	4.00-9.00
Hemoglobin	g/dL	9.1	12.0-16.0
Platelet count	×10^3^/μL	228	150-450
Total bilirubin	mg/dL	1.2	0.2-1.2
Aspartate aminotransferase	U/L	24	13-33
Alanine aminotransferase	U/L	21	8-42
Lactate dehydrogenase	U/L	393	124-222
Alkaline phosphatase	U/L	87	38-113
Gamma-glutamyl transferase	U/L	123	11-58
Amylase	U/L	149	33-120
HbA1c	%	4.8	4.6-6.2
Total protein	g/dL	6.1	6.6-8.7
Albumin	g/dL	2.8	3.4-4.8
Albumin/globulin ratio	ratio	0.8	1.2-2.0
Blood urea nitrogen	mg/dL	8	5.0-23
Creatinine	mg/dL	0.53	0.36-1.06
Estimated glomerular filtration rate	mL/min/1.73 m²	118.9	≥90
Soluble interleukin-2 receptor	U/mL	800	122-496
C-reactive protein	mg/dL	8.61	0.00-0.30

**Figure 1:**
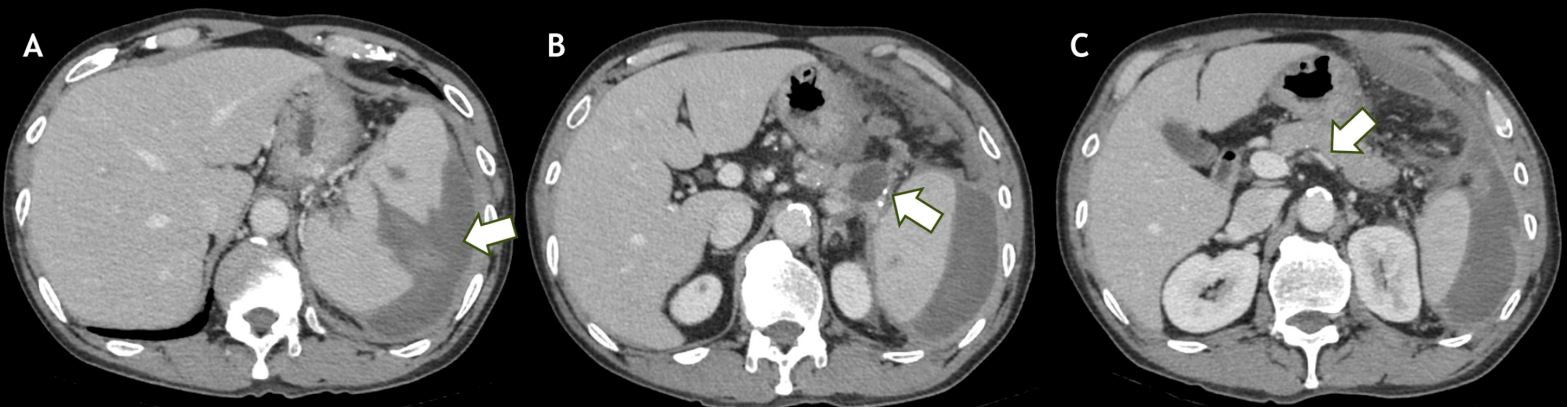
Contrast-enhanced axial computed tomography findings on admission (A) Axial contrast-enhanced computed tomography image demonstrating splenic laceration with associated perisplenic hematoma. (B) Axial computed tomography image showing a cystic lesion in the pancreatic tail adjacent to the splenic hilum. (C) Axial computed tomography image demonstrating narrowing/occlusion of the splenic vein anterior to the abdominal aorta. No active contrast extravasation is identified.

Contrast-enhanced computed tomography of the chest and abdomen demonstrated a large splenic laceration with surrounding hematoma and fluid collection. Compared with the computed tomography performed approximately four hours earlier at the referring hospital, there was no significant interval change. No active contrast extravasation from the spleen was identified. Splenomegaly, a pancreatic cystic lesion, and narrowing of the splenic vein were also noted (Figure 1). Although classic advanced morphologic features of chronic pancreatitis, such as marked main pancreatic duct dilatation or obvious parenchymal calcification, were not prominent on the presented images, a cystic lesion in the pancreatic tail adjacent to the splenic hilum and narrowing/occlusion of the splenic vein were identified.

**Figure 2:**
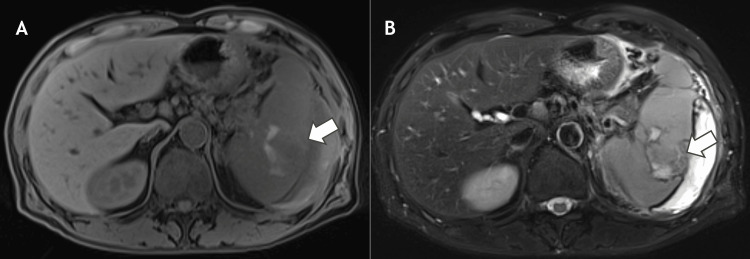
Magnetic resonance imaging findings of the spleen (A) T1-weighted axial image and (B) T2-weighted axial image demonstrating heterogeneous signal intensity within the splenic parenchyma (arrows), raising concern for a possible neoplastic process.

Magnetic resonance imaging demonstrated no substantial increase in the size of the splenic hematoma. However, heterogeneous signal intensity within the splenic parenchyma raised concern for a possible neoplastic lesion, and a neoplastic process could not be excluded (Figure 2).

Because no active bleeding was identified and the patient remained hemodynamically stable, conservative management was initiated. Additional investigations for infectious and neoplastic etiologies were performed after admission. Although no definitive evidence of malignancy was obtained, a malignant lesion could not be ruled out completely. In addition, given the mechanism of splenic injury and the possibility of delayed hemorrhage during nonoperative management, definitive surgical intervention was considered appropriate.

**Figure 3:**
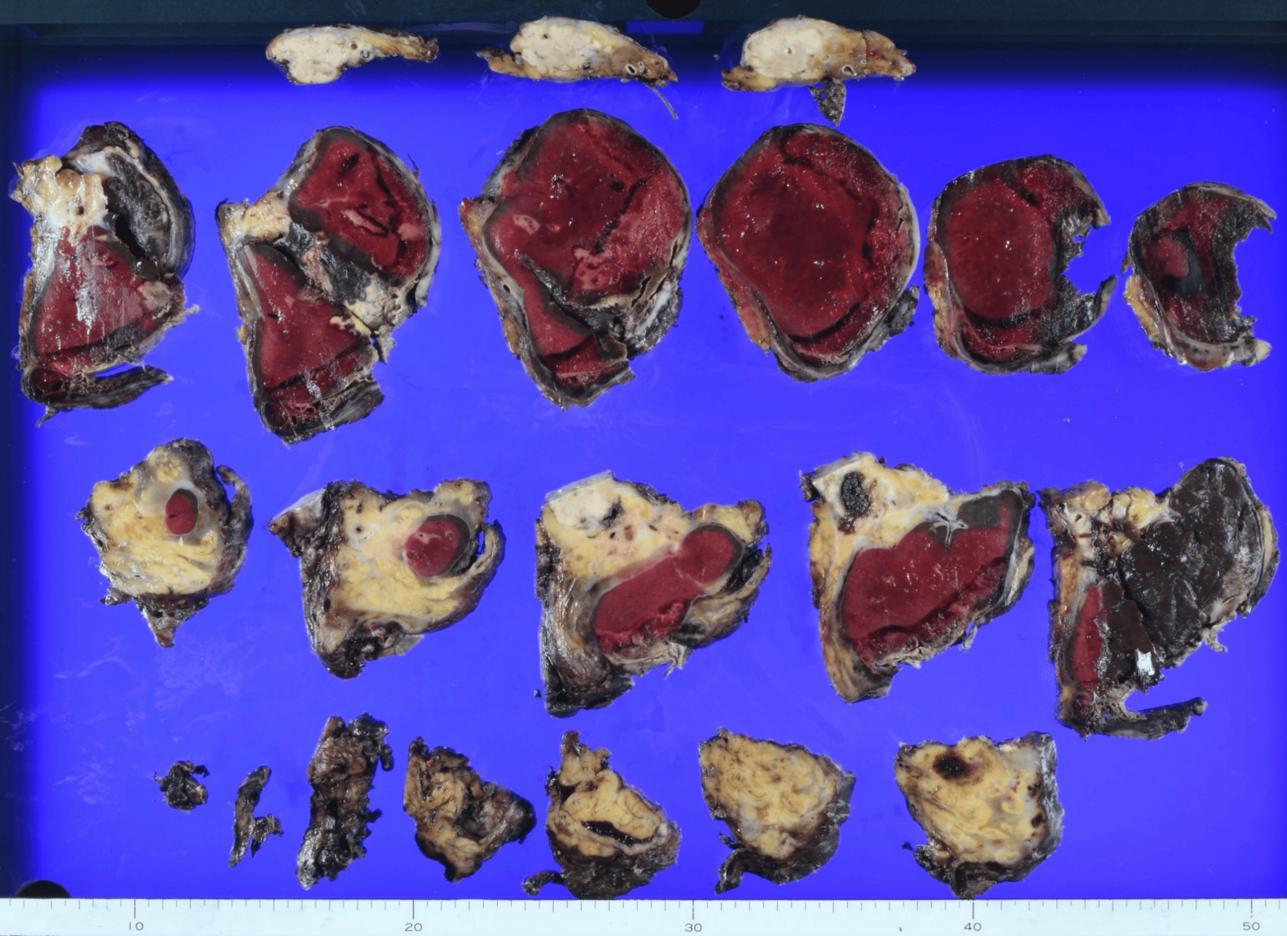
Gross appearance of the resected specimen The resected spleen shows a large splenic laceration with extensive hematoma formation. The specimen was obtained by splenectomy with distal pancreatectomy and partial diaphragmatic resection.

On hospital day 9, the patient underwent open splenectomy with distal pancreatectomy and partial diaphragmatic resection. Intraoperatively, there were dense inflammatory adhesions extending from the pancreatic tail to the spleen and surrounding tissues, as well as dense adhesion to the diaphragm. The pancreas was indurated, consistent with chronic inflammatory change. Isolated splenectomy was not feasible; therefore, combined resection of the distal pancreas and part of the diaphragm was performed. The operative time was eight hours and 36 minutes, and blood loss was 5,903 mL. Macroscopic examination of the resected specimen revealed a large splenic laceration with hematoma formation (Figure 3).

**Figure 4:**
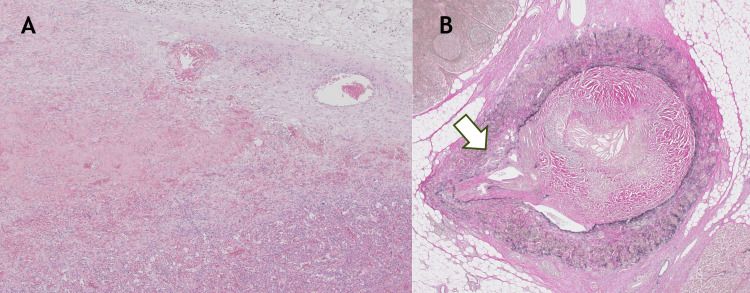
Histopathological findings of the resected specimen (A) Hematoxylin and eosin staining, ×2, demonstrating extensive hemorrhage and congestion within the splenic parenchyma. (B) Elastica van Gieson staining, ×2, showing an organized thrombus within the splenic vein with associated inflammatory change and partial disruption of the vascular wall (arrow). These findings support atraumatic splenic rupture secondary to splenic vein thrombosis with occlusion associated with chronic pancreatitis.

Histopathological examination showed no evidence of malignancy. The resected distal pancreas was pathologically diagnosed as chronic pancreatitis. Histological examination demonstrated fibrosis, mild chronic inflammatory cell infiltration, fat necrosis, pseudolobular formation, and focal acute inflammation, consistent with acute-on-chronic pancreatitis. In the splenic hilar region and surrounding tissue, partial loss of the splenic vascular wall, organized thrombus within the splenic vein, and marked splenic congestion were also identified (Figure 4). No malignant lesion was identified in the resected pancreas or spleen. These findings supported the diagnosis of atraumatic splenic rupture caused by increased intrasplenic pressure secondary to splenic vein thrombosis with occlusion associated with chronic pancreatitis. The postoperative course was uneventful, and no major postoperative morbidity occurred. The patient was discharged on postoperative day 15.

## Discussion

This case is clinically important because it illustrates a rare but pathophysiologically plausible form of pancreatitis-related atraumatic splenic rupture that was ultimately confirmed by histopathology. In the systematic review by Renzulli et al., 632 publications comprising 845 patients showed that most atraumatic splenic ruptures were pathological rather than idiopathic, that total splenectomy was performed in 84.1% of patients, and that mortality was 12.2%. Moreover, splenomegaly, age over 40 years, and neoplastic disease were associated with worse outcomes [1]. This patient was older than 40 years and had splenomegaly, both of which underscored the importance of careful diagnostic and therapeutic decision-making.

Among patients with chronic pancreatitis, splenic complications are uncommon but well-documented. In a prospective medical-surgical series of 500 consecutive patients with chronic pancreatitis, Malka et al. found splenic complications in 11 patients (2.2%), including four cases of splenic rupture. These complications occurred early in the course of the disease and were significantly associated with pancreatic tail necrosis, distal pseudocyst formation, and splenic vein occlusion. In the subgroup of patients with both distal pseudocyst and splenic vein occlusion, the prevalence of splenic complications rose to 18% [2]. In this patient, the combination of chronic pancreatitis, a pancreatic tail cystic lesion, and radiologic splenic vein narrowing with occlusion represented a biologically and clinically credible high-risk background.

An additional point requiring clarification in this case is the basis for the diagnosis of chronic pancreatitis. Although classic advanced morphologic imaging features of chronic pancreatitis, such as marked main pancreatic duct dilatation or obvious parenchymal calcification, were not prominent on the presented cross-sectional images, the diagnosis was supported by multiple other findings. The patient had a prior history of chronic pancreatitis related to alcoholic pancreatitis, serum amylase was mildly elevated at admission, and intraoperative examination demonstrated pancreatic induration consistent with chronic inflammatory change. Furthermore, histopathological examination of the resected distal pancreas showed fibrosis, mild chronic inflammatory cell infiltration, fat necrosis, pseudolobular formation, and focal acute inflammation, consistent with acute-on-chronic pancreatitis. HbA1c was 4.8%, without clear evidence of endocrine dysfunction at presentation, whereas formal pancreatic exocrine function testing had not been performed before surgery. Therefore, in this case, the diagnosis of chronic pancreatitis was based on the prior clinical history together with intraoperative and pathological findings, rather than on imaging findings alone. A microscopic pancreatic histology image was not available for inclusion in this report, which should be acknowledged as a limitation.

The mechanism of rupture in the present case appears to have been venous outflow obstruction rather than direct pseudocyst penetration into the spleen. The pathological findings of organized thrombus within the splenic vein, marked splenic congestion, and partial loss of the splenic vascular wall strongly support progressive venous obstruction with increased intrasplenic pressure as the main mechanism of rupture. This interpretation is consistent with prior reports suggesting that splenic vein thrombosis can cause congestion, impaired microcirculation, infarction, parenchymal fragility, and eventual rupture [4,7-10]. Zhou et al. similarly described atraumatic splenic rupture precipitated by splenic vein thrombosis and emphasized venous congestion as a key pathophysiological link [8]. This case adds histopathological support to this mechanism in the setting of chronic pancreatitis.

The broader literature on pancreatitis-induced splenic vein thrombosis also helps contextualize the management strategy. Heider et al. reported that the natural history of pancreatitis-induced splenic vein thrombosis is often relatively benign with respect to gastric variceal bleeding, with an overall hemorrhage rate of approximately 4%, and concluded that routine prophylactic splenectomy is not indicated for splenic vein thrombosis alone [5]. Agarwal et al. likewise showed that splenic vein thrombosis is not rare in chronic pancreatitis, reporting an incidence of 22%, although clinically significant variceal bleeding occurred in only a minority of patients [6]. These data are important because they show that splenic vein thrombosis itself does not automatically mandate surgery. However, this patient had already developed atraumatic splenic rupture, making the clinical situation fundamentally different from isolated asymptomatic splenic vein thrombosis.

Imaging was central to both diagnosis and treatment planning. Fishman et al. described splenic involvement in pancreatitis as rare, with an estimated frequency of 1-5%, and highlighted the computed tomography spectrum of intrasplenic pseudocyst, abscess, hemorrhage, infarction, rupture, and vascular injury [4]. In this case, contrast-enhanced computed tomography clearly demonstrated splenic laceration, perisplenic hematoma, and fluid collection while also showing splenomegaly, a pancreatic cystic lesion, and splenic vein narrowing. Magnetic resonance imaging further demonstrated heterogeneous splenic signal intensity, which raised concern for a possible neoplastic lesion. Thus, cross-sectional imaging not only detected the rupture but also influenced the decision to proceed with surgery because malignancy could not be confidently excluded.

The optimal management of atraumatic splenic rupture depends on hemodynamic status, suspected etiology, imaging findings, and the need for definitive diagnosis [1,7-10]. Several reports have shown that conservative treatment or splenic artery embolization can be considered in selected hemodynamically stable patients [7,8]. In particular, Zhou et al. reported successful splenic artery embolization as a bridge to delayed splenectomy in a patient with splenic vein thrombosis-related atraumatic splenic rupture [8]. At the same time, Nadaraja et al. emphasized that splenectomy may still be justified even in hemodynamically stable patients because histological examination can clarify the underlying diagnosis and because a substantial proportion of atraumatic splenic ruptures are associated with pathological spleens, including malignancy [9]. This principle was especially relevant in this patient, in whom magnetic resonance imaging did not confidently exclude a neoplastic process. In addition, the extensive inflammatory adhesions around the pancreatic tail and spleen made isolated splenectomy technically impossible, necessitating distal pancreatectomy and partial diaphragmatic resection.

Compared with previously published cases, this case is notable for the concordance between clinical history, operative findings, and pathology. Martelo et al. reported a similar Cureus case in which chronic pancreatitis was considered the cause of atraumatic splenic rupture, with splenic vein thrombosis thought to be the precipitating factor [10]. However, the present case provides particularly clear pathological evidence linking chronic pancreatitis, splenic vein thrombosis with occlusion, severe congestion, and rupture. This strengthens the argument that splenic vein thrombosis is not merely an incidental imaging finding in some pancreatitis-related splenic ruptures but may instead represent the dominant causal mechanism.

Taken together, the available evidence suggests a nuanced treatment paradigm. For isolated pancreatitis-induced splenic vein thrombosis without bleeding, routine prophylactic splenectomy is not supported by cohort data [5,6]. For atraumatic splenic rupture, however, especially when splenomegaly is present, malignancy cannot be excluded, or re-bleeding is a concern, operative management remains well-justified and is consistent with the dominant treatment pattern reported in the systematic review by Renzulli et al. [1]. In this context, the chosen management strategy was appropriate for both definitive diagnosis and definitive treatment.

## Conclusions

A rare case of atraumatic splenic rupture secondary to splenic vein thrombosis with occlusion associated with chronic pancreatitis was encountered. The diagnosis of chronic pancreatitis in this case was supported by the prior history of alcoholic pancreatitis, intraoperative pancreatic induration, and pathological findings in the resected distal pancreas. Histopathological examination also demonstrated organized splenic venous thrombosis and marked splenic congestion without evidence of malignancy, supporting venous outflow obstruction with increased intrasplenic pressure as the mechanism of rupture.

In patients with chronic pancreatitis who present with left upper quadrant pain, splenic complications should be considered promptly. When the diagnosis remains uncertain, when re-bleeding is a concern, or when pathological confirmation is required, splenectomy may be an appropriate therapeutic and diagnostic option.
